# Surface and deep learning: a blended learning approach in preclinical years of medical school

**DOI:** 10.1186/s12909-024-05963-5

**Published:** 2024-09-19

**Authors:** Mei Li Khong, Julian Alexander Tanner

**Affiliations:** 1 School of Clinical Medicine, Li Ka Shing Faculty of Medicine, The University of Hong Kong, Hong Kong Special Administrative Region, P.R. China; 2 School of Biomedical Sciences, Li Ka Shing Faculty of Medicine, The University of Hong Kong, Hong Kong Special Administrative Region, P.R. China

**Keywords:** Blended learning, Digital education, Online learning, Traditional learning, Face-to-face learning, Surface learning, Deep learning, R-SPQ-2F

## Abstract

**Background:**

Significant challenges are arising around how to best enable peer communities, broaden educational reach, and innovate in pedagogy. While digital education can address these challenges, digital elements alone do not guarantee effective learning. This study reports a blended learning approach integrating online and face-to-face components, guided by the Student Approaches to Learning framework.

**Methods:**

This study was carried out investigating learning in first and second year medical students over two academic years, 2019/20 and 2020/21. We evaluated: (1) comparison of students engaged with blended learning and traditional learning; and (2) student learning engaged with blended learning approach over a two-year preclinical curriculum. A revised two-factor study process questionnaire (R-SPQ-2F) evaluated students’ surface/deep learning before and after an academic year. Learning experience (LE) questionnaire was administered over the domains of learning engagement, and outcomes of learning approach. In-depth interviews were carried out to understand the context of students’ responses to the R-SPQ-2F and LE questionnaires.

**Results:**

The R-SPQ-2F analysis indicated first year students maintained deep learning but second year students became neutral across the academic year, regardless of learning approach, with workload contributing to this outcome. R-SPQ-2F sub-scales showed that students engaged with blended learning maintained an intrinsic interest to learning, as compared to traditional learning which led to surface learning motives. The LE questionnaire showed students engaged with blended learning had deeper subject interest, and more positive perceptions of workload, feedback, and effectively developed skills and knowledge. However, peer interactions from blended learning were significantly lacking. In-depth interviews revealed that the flexibility and multi-modality of blended learning enabled learning, but the best use of these features require teacher support. Online interactions could be cultivated through intentional institutional efforts.

**Conclusions:**

This study highlights the importance of designing blended learning that leverages technology-enabled flexibility while prioritising collaborative, learner-centred spaces for deep engagement and knowledge construction.

**Supplementary Information:**

The online version contains supplementary material available at 10.1186/s12909-024-05963-5.

## Background

Student learning is moving from the lecture hall to online environments where rich peer-to-peer learning communities can be fostered, where impact can propagate to multiple campuses globally, and where innovative delivery methods can be developed [1, 2]. Educators can easily update teaching content, track students’ learning interest on each topic via learning analytics and obtain feedback through online discussion. Students can freely arrange their learning and style according to their own needs [3]. However, is this more of a vision than reality?

Digital education encompasses a wide spectrum of approaches from PowerPoint presentations with audio voice-over, through video lectures and annotation of these videos, direct links to other Web contents, simulations, dashboards, virtual worlds, gamified environments, virtual communities, and Massive Open Online Course (MOOC) [4]. The learner is provided with different types of media and varied approaches for learning course content. Web-based instruction can use less classroom time but increase students’ ability to attain similar educational goals [3]. The world’s top universities, including Stanford, Harvard and MIT, are all embracing digital education, with widespread use across many programmes both on and beyond the campus [5].

In an Asian context and within Hong Kong, Lam and colleagues put into perspective how students’ technology experience shapes the effectiveness of digital education [6]. The use of technology is an integral part of students’ everyday lives for networking and communication. However, Lam and colleagues reported that students’ experience in using digital education strategies were mostly limited to simple access to course information in learning management systems (e.g. Moodle) and relevant websites. Despite the lack of experience in digital education strategies, students favour the use of an online platform for peer-peer and teacher-student interactions and feedback processes [6]. Here, we see opportunity to engage students’ digital experiences in their daily lives within an educational context.

Studies in health professions education (HPE) have established that online learning can be at least as effective as traditional face-to-face learning [7, 8]. However, there is still much to learn about how to optimise the use of digital education in HPE [9, 10], and few have developed digital education in line with a learning theory [11]. We considered different course design models (blended, hybrid, hyflex) to optimise online and face-to-face components for medical education [12–14]. The hybrid model replaces much of the face-to-face time with synchronous or asynchronous online interactions. The hyflex model combines both ‘hybrid’ and ‘flexible’ to offer in-person interactions that are synchronously or asynchronously online for flexibility through multi-modal participation. However, a medical programme requires learners to conceptually learn and apply through practice and solving of real-life problems. Therefore, we considered the blended model as most suitable in enabling both online and face-to-face learning modalities to be integrated cohesively. Online resources and activities are meant to complement face-to-face time.

The literature also proposed blended learning course designs in MOOC environments for large cohorts in higher education [15–17]. Students watched video lectures on their own, practised problems as self-assessment, and further met the instructors during face-to-face class time to discuss concepts presented in lecture videos. However, these approaches still follow a teacher-centred model where the course was scheduled, led by the instructor, and did not enable students to engage in a self-organised learning experience.

We hypothesised that an evidence-based, blended learning approach which integrates both online and face-to-face learning is relevant to medical education. This paper reports the development and evaluation of a blended learning approach in preclinical years of medical school which promotes students’ self-organised deep learning. We aimed to study the impact of this approach on the learning process and experience of learners, as well as to develop informed recommendations for effective instructional design.

### Conceptual framework

We utilised the Student Approaches to Learning (SAL) theory as a framework [18, 19] to evaluate our blended learning approach in the curriculum. SAL posits that both teachers and students are jointly responsible for the learning outcome – the teacher for structuring the enabling conditions, the learner for engaging them. Factors influencing learning include student characteristics (prior knowledge, ability, preferred learning approaches) and teaching context (including content, methods of teaching and assessment, institutional climate, and so on) [20].

We applied the SAL framework to identify an optimal learning approach in preclinical education. Many scholars have expanded the concept of deep approach (DA) to learning, positing that it is a process of organising, synthesising, and transferring concepts. In contrast, surface approach (SA) to learning emphasises rote learning, with the aim of acquiring enough knowledge to complete tasks or pass courses [21]. Surface approaches indicate issues in teaching or assessment methods that need addressing. The result of our study may guide medical schools in their efforts to best support learning with approaches that combine online and face-to-face learning.

## Methods

### Study context

The Bachelor of Medicine and Bachelor of Surgery (MBBS) programme at HKU provides comprehensive conceptual and applied learning of basic sciences in the preclinical years (Years 1 and 2) of medical school, before learners proceed to clinical practice. The MBBS programme underwent a major modernisation where digital education took on a far more prominent role in these preclinical years. We saw challenges including greater student numbers (to over 300 students per year), increased diversity in student educational background and learning approach, information overload, changing societal needs and expectations, and disruptive innovations.

We therefore modernised our instructive methods, using a blended learning approach by incorporating online lecture videos into approximately 50% of the curriculum coupled to live interactive sessions and formative assessments containing built-in feedback. Conceptual lectures were converted to online lecture videos, while lectures that required reflective thinking and immediate student-teacher discourse remained as live lectures. As of any digital education programme, there is a challenge of ensuring student engagement to the content, and ensuring connections amongst peers and instructors [22]. Therefore, we included interactive online forum discussions within the online learning system to enable peer-peer and teacher-student interactions. Our new student intake is predominantly composed of undergraduate students transitioning from local or international secondary schools. To help these students adapt to tertiary education learning, curriculum design is oriented towards providing structure and support to student learning.

**Fig. 1:**
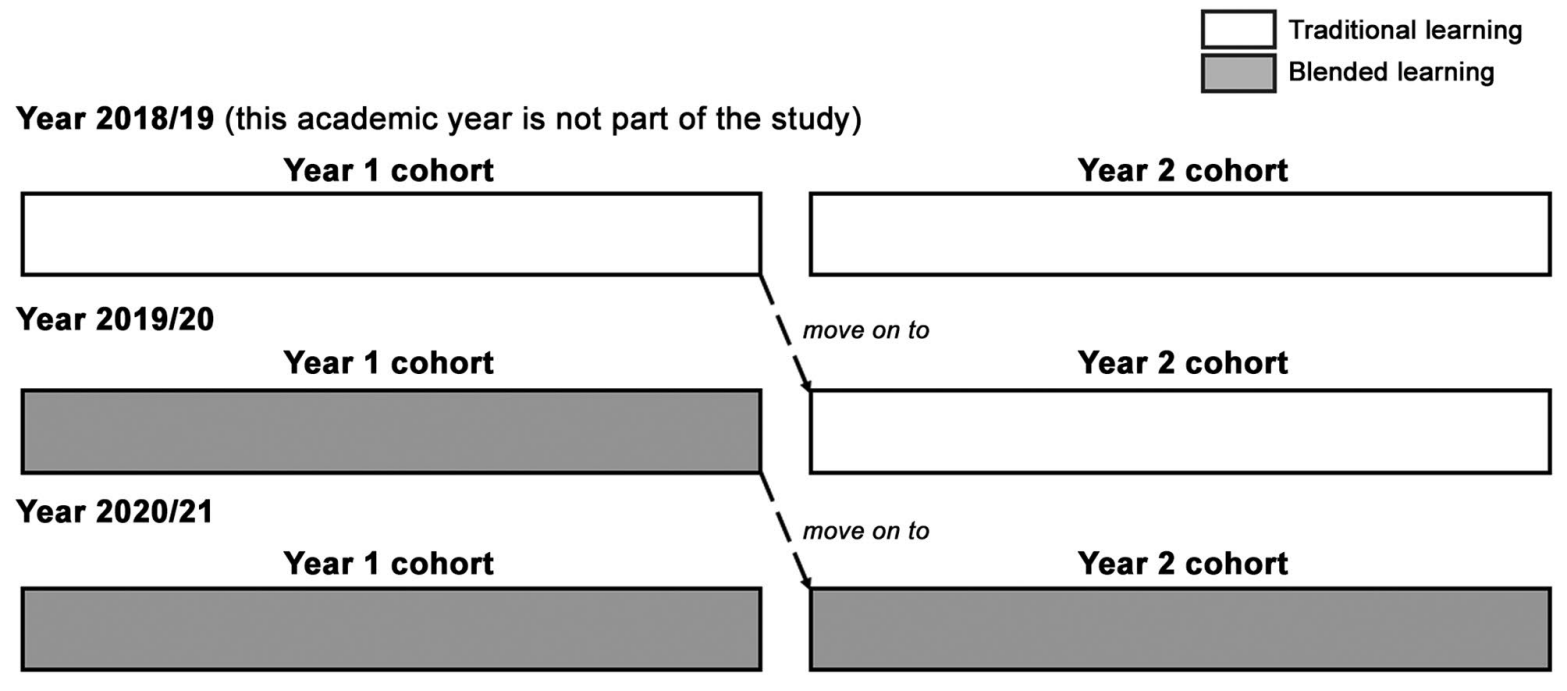
Modernisation phases in preclinical years of MBBS

The modernisation initiative took place in phases. Figure 1 illustrates the phases of modernisation. Of note, the coronavirus disease 2019 (COVID-19) pandemic and restrictions in January 2020 onwards did not extensively affect the implementation of blended learning approach but instead propelled its active implementation [23, 24]. During social distancing restrictions, the active learning sessions and traditional classroom learning were live sessions held through virtual platforms (e.g. Zoom), under the moderation of technical experts.

This paper reports on the empirical data collected from the wider project that aimed to develop blended learning into the medical curriculum. We used a mixed-methods approach which triangulates student learning approach, process and experience.

### Participants and study design

Following ethical and institutional approvals, all MBBS Year 1 and Year 2 students (in academic years 2019/2020 and 2020/2021) were invited via email to participate in multiple phases.

This study compares students engaged with blended learning and traditional learning in the preclinical MBBS curriculum across academic years 2019/2020 and 2020/2021. A revised two-factor study process questionnaire (R-SPQ-2F) was used to assess students’ surface/deep learning at the start and end of the year. Learning experience (LE) questionnaire was administered (11 items) over the domains of learning engagement (6 items), and outcomes of learning approach (5 items). In-depth individual interviews were performed to understand the context of students’ responses in terms of learning approach, process, and experience.

### Data collection and analysis

Data collection was carried out by author MLK who was independent of MBBS students’ academic assessment. All students’ personal identifiers were removed by MLK before passing onto other investigator(s) of this study for data analysis and storage. Students’ responses to the survey did not have any impact on their study performance and assessments. We used a mixed-method study incorporating evaluation for student and learning described by Sarsa and Escudero [4], Biggs and colleagues [20], as well as, Kember and Ginns [25]. According to reviews of Randolph’s study [4], such mixed-method approaches allow a holistic and valid view of a phenomenon by interpretation from multiple perspectives. Quantitative studies provide explanations through an objective manner, while qualitative evaluation allowed deeper understanding of the subject matter.

For the R-SPQ-2F, pre- and post-course data was collected in every cohort of the intervention and control groups, respectively. Pre- and post-course validated questionnaires were disseminated online and comprised a set of questions (20 questions) to assess students’ learning approaches and process [20]. The intervention group is composed of students who experienced the blended learning approach. As all students experienced equivalent blended learning in Year 1 (2019/20) and Years 1 and 2 (2020/21), we consider the Year 2 students in 2019/20 (who experienced the traditional classroom learning throughout their preclinical years) as control group (Fig. 1). Subjects remained anonymous prior to analysis. The data was computed as described in Additional file 1, analysed in accordance to scales and sub-scales described in Table 1 [20]. Data analysis was done using descriptive statistics and statistical associations.

**Table 1:** R-SPQ-2F: dimensions, motives and strategies

	Surface	Deep
Motive	Fear of failure	Intrinsic interest
Strategy	Narrow target, rote learn	Maximise meaning

For the post-course LE questionnaire (Additional file 2), we probed students’ perceived experience of the blended learning approach on a post-course online survey basis. Evaluation of students’ experience were formatted in the questionnaire following the model of Kember and Ginns [25]. A 5-point Likert scale was used as shown: (1 – Strongly disagree; 2 – Disagree; 3 – Neither agree nor disagree; 4 – Agree; 5 – Strongly agree). Subjects remained anonymous prior to analysis. Data analysis was done using descriptive statistics and statistical associations. Both intervention and control group setup apply for this quantitative data evaluation.

For the post-course in-depth individual interviews, open-ended questions with constructs guided by the results of the prior questionnaires were included (Additional file 3). The aim was to assess the context of student responses in the earlier questionnaires. Categories were not pre-defined but rather emerged from data. All students who participated in prior questionnaires were invited for interview on voluntary basis. Interviews continued until saturation of themes (when all concepts are repeated multiple times without new themes emerging) [26, 27]. Interviews were audio-recorded or text-recorded (if participant did not consent to audio recording). Subjects remained anonymous prior to analysis. We followed good practices of data analysis from Gardner and colleagues [28, 29]. Audio recordings were transcribed verbatim and transcripts were sent to all student participants for verification. Then, the free responses were analysed based on grounded theory by at least two independent researchers to confirm the themes identified, refine themes and reach a consensus.

## Results

All students in Years 1 and 2 cohorts across academic years 2019/20 and 2020/21 were eligible for the study. Subjects from the intervention group completed the course through a blended learning approach, while subjects from the control group completed the course through a traditional classroom learning approach.

### Learning approach and process

Of all the invited and eligible students (approximately 250 students in each cohort), those who consented and completed the R-SPQ-2F are as follows: 24 from Year 2 (2019/20) – control; 65 from Year 1 (2019/20) – intervention; 21 from Year 2 (2020/21) – intervention; 36 from Year 1 (2020/21) – intervention.

**Fig. 2:**
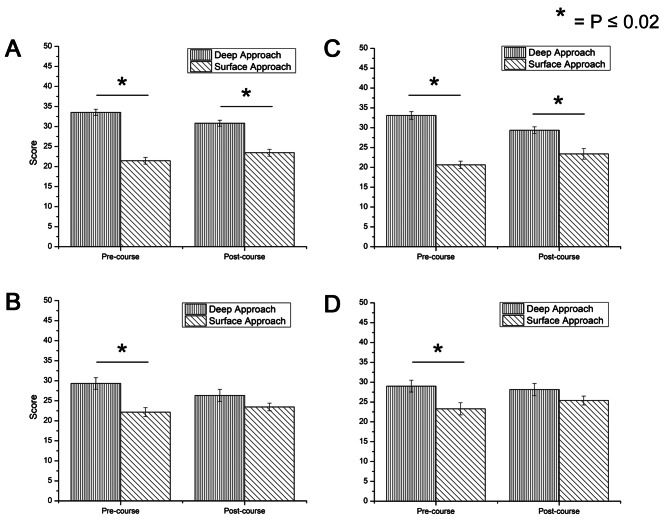
Surface/Deep learning across cohorts with scales Deep Approach (DA) and Surface Approach (SA). **(A)** MBBS Year 1 (2019/20) (intervention); **(B)** MBBS Year 2 (2019/20) (control); **(C)** MBBS Year 1 (2020/21) (intervention); **(D)** MBBS Year 2 (2020/21) (intervention). Error bar represents standard error of mean (SEM)

**Fig. 3:**
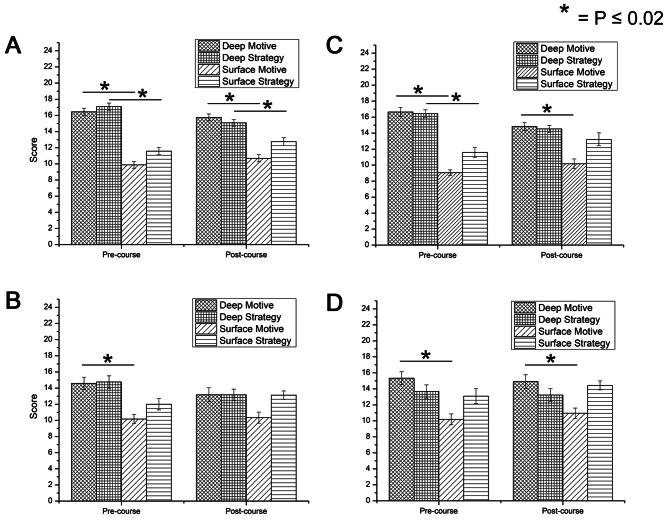
Surface/Deep learning across cohorts with sub-scales Deep Motive (DM), Deep Strategy (DS), Surface Motive (SM), and Surface Strategy (SS). **(A)** MBBS Year 1 (2019/20) (intervention); **(B)** MBBS Year 2 (2019/20) (control); **(C)** MBBS Year 1 (2020/21) (intervention); **(D)** MBBS Year 2 (2020/21) (intervention). Error bar represents standard error of mean (SEM)

Figure 2A and C show two cohorts of Year 1 students, that both engaged with blended learning approach. Data showed that a deep approach (DA) to learning was maintained across the academic year. Figure 3A and C further demonstrate that Year 1 students begun with significantly deeper motive and deeper strategy. The 2019/20 Year 1 cohort remained with a significantly deeper motive and strategy by the end of the course. The 2020/21 Year 1 cohort remained with a significantly deeper motive by the end of the course but concurrently became neutral in terms of deep or surface strategy (*p* > 0.02).

On the other hand, Fig. 2B and D demonstrated that Year 2 students started off with a significantly deep approach to learning but became neutral (*p* > 0.02) by the end of the academic year, regardless of whether traditional or blended learning was employed. We then analyse Fig. 3B and D. For the 2019/20 Year 2 cohort which experienced traditional learning, they started off with a significantly deeper motive, but was neutral in deep or surface strategy (*p* > 0.02). By the end of the course, however, students became neutral in both motive and strategy (Fig. 3B). For the 2020/21 Year 2 cohort which experienced blended learning, they similarly started off with significantly deeper motive but was neutral in deep or surface strategy (*p* > 0.02). By the end of the course, the trend remains the same (Fig. 3D), suggesting that those who underwent blended learning kept an intrinsic interest to learning compared to those who underwent traditional learning.

### Learning experience

For the LE questionnaire, eligible study subjects used for comparison were MBBS Year 2 students from 2019/20 (control) and MBBS Year 2 students from 2020/21 (intervention). This enables direct comparison between two cohorts of the same year of study with similar curriculum content, but with different learning approaches. Of all the invited eligible students, 16 from control group and 24 from intervention group consented and completed the LE questionnaire.

**Fig. 4:**
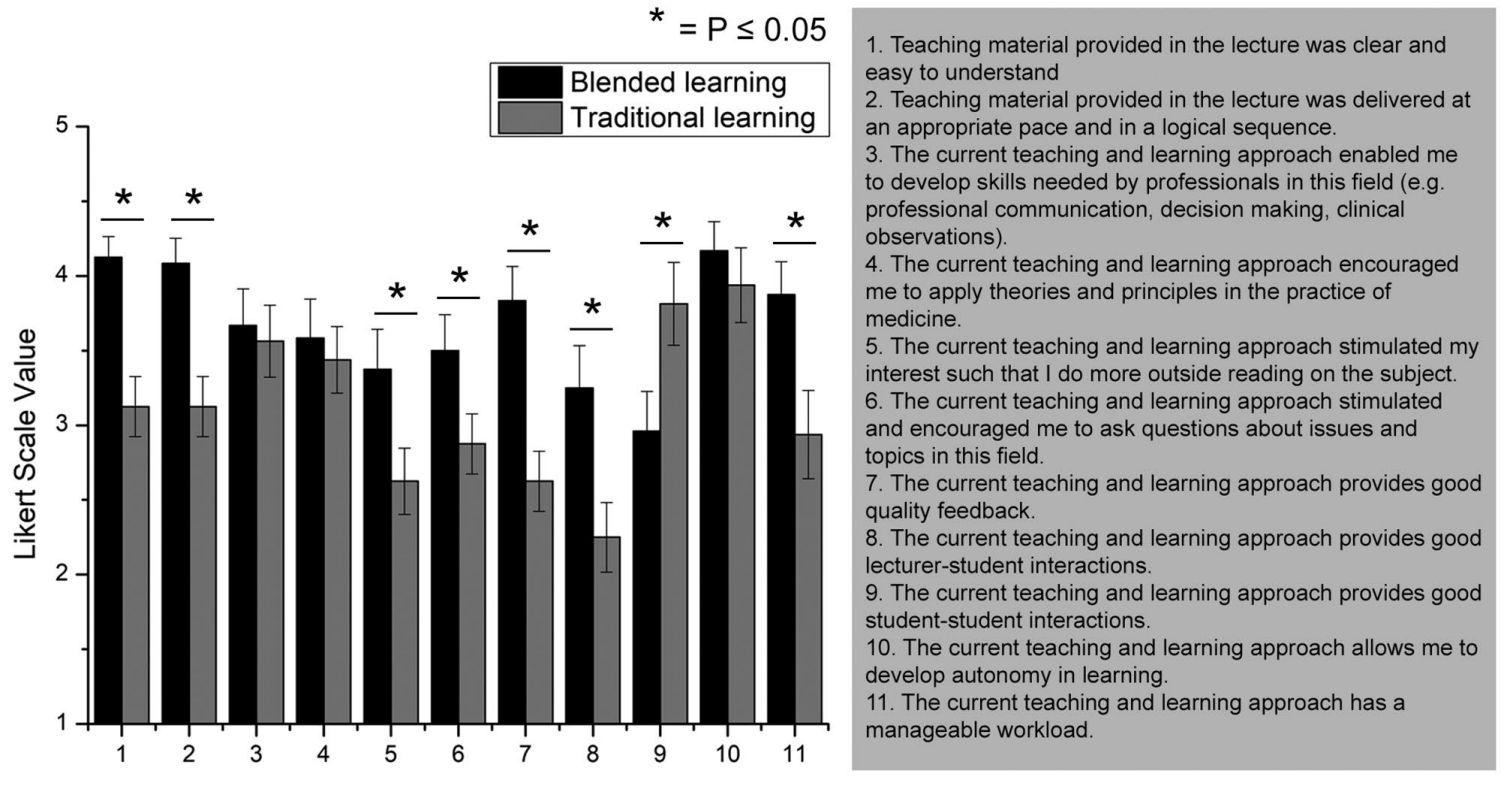
Comparison of learning experiences between blended learning and traditional learning. Error bar represents standard error of mean (SEM)

The LE questionnaire showed that students doing blended learning have deeper interest in their subject (items 5 and 6; *p* ≤ 0.05); a more positive perception of the delivery of lectures (items 1 and 2; *p* < 0.005), learning feedback (item 7; *p* < 0.005), and workload (item 11; *p* ≤ 0.05) as compared to students who did traditional learning. Both groups’ perceived development of skills and knowledge were similar for both learning approaches (items 3 and 4; *p* > 0.05). Perception of learning autonomy also remained similar across both approaches (item 10; *p* > 0.05). However, peer interactions were perceived to be lacking in a blended learning environment (item 9; *p* ≤ 0.05) even though lecturer-student interactions improved in such environment (item 8; *p* ≤ 0.05).

### Triangulation of blended learning approach, process, and experience

Data from LE questionnaire further informed the semi-structured constructs of the in-depth, individual interviews. The context of students’ responses in both R-SPQ-2F and LE questionnaire was further explored through the interviews. The total sample includes 22 participants who encountered the blended learning approach.

Additional file 4 consolidates the participants’ characteristics according to SAL theory, which describes factors affecting learning include student characteristics such as prior knowledge and experiences, learning ability, and preferred learning approaches.

#### Blended learning accommodates learner needs and differences through multi-modalities

Our curriculum comprised of online lecture videos with accompanying self-assessment questions, online forums, and live interactive sessions. With combined online and face-to-face learning, students reported that the multi-modalities met the needs of different types of learners. At the most basic format of online learning, video captions and visual aids (e.g. cursor annotations, diagrams, video snippets) were available across all online lecture videos.

The textual captions were useful for textual learners by providing comprehension of technical terminologies and content, as well as ease of notetaking. Visual aids were found to be particularly important for visual learners or subjects that require visual learning. Some important visual aids highlighted were embryology animations; histological, microbiological, pathological specimen viewing.

*[Online lecture videos] allows us to better jot notes because video clips are very clear and have high quality and it allows us to easily take a screenshot of the video for our notes*,* and I think it’s something that live lectures cannot do. I think it’s also very useful for us to have such comprehensive captions under each video because it is normal for students to not understand some new words*,* especially some technical terms. I think the captions allow us to capture the technicality of new terms very easily.* BL21.

*I think a lot of my peers will agree that embryology benefit from having figures and diagrams and animations to show us how everything folds together and how other tissues grow because it’s difficult to understand how everything is positioned.* BL03.

Self-assessment questions following every online lecture videos enabled immediate and personalised feedback. Students were able to check their conceptual understanding, clarify learning aims, clear misconceptions, and practise for exams. In-video questions were further appreciated by several students causing them to think deeper while going through the content.

*We can check our understanding after watching a video and I also appreciate how there is some individualised feedback for those questions. There will be explanations saying why an option is correct or incorrect. I think this allows immediate feedback for my learning… how I get things wrong and how I can improve myself. And another good thing about this is that we can understand what is expected of us.* BL06.

Online forums were particularly useful for inquiring minds to freely raise post-lecture questions, engage in active discussions, and/or build connections. At the same time, it provided equitable learning for those who preferred to read the ongoing discussions to learn from it.

…*the lecturer gives a relatively fast instant reply*,* so it can really instantly address student’s problem. Since it is on the online forum*,* students won’t just ask any random or poorly constructed questions there. They will ask some really constructive and specific questions….*

*And sometimes I find looking at others’ questions as well as lecturers’ responses very useful. They act like additional information for me*. BL05.

*In the forum*,* we see people discussing about different questions and some of them might be questions of your own. Previously*,* people would just individually email lecturers to ask questions*,* but now it’s shared. So*,* I really like how everything is visualised and open to public. So*,* everyone can also think about it or also get the answers….* BL08.

Students generally agreed that blended learning is a good approach for medical education. Online lecture videos were most suitable for learning facts, and this complements the application of learning through live sessions like interactive sessions.

*I think it is possible to do all lectures in [an online format]…. The value of face-to-face classes will be the hands-on nature of the session or application of skills and knowledge. In face-to-face classes*,* we can talk to our peers to exchange knowledge*,* correct each other’s misunderstanding*,* and we can also communicate with teachers to ask them questions or better understand the materials. These reinforce your memory.* BL06.

*We get challenged by the interactive sessions and you’re sort of taken back by what you don’t know and I think it’s exciting.* BL12.

*In live sessions*,* professors include some case study and I find it really stimulating. We apply our knowledge into these cases and see whether we can solve the problem.* BL15.

#### Flexibility of online learning enables deep learning

Students found that the online component in the blended learning approach enables them to control how and when they study. They could adjust their learning pace to do intermediate research on core topics for deeper understanding. Online learning also permits more time to consolidate better notes and diagram annotations while watching the videos. Nearing examination period, students can review the lecture videos for effective revision.

*You can choose when and where to watch the lecture videos based on your own schedule. I work better at night and can absorb more knowledge…. By watching the video at my own pace*,* I get to pause and search in the middle of the lectures. It’s really helpful and it’s actually more efficient for my personal learning. Watching online lecture videos with my peers is also another good thing. We get to pause and really think and discuss immediately*,* but while in lecture we might not get that opportunity to do so because it is just impolite to do it. Also*,* in online lecture videos*,* because we get the videos kind of chopped up in chunks*,* so it’s also a good way of organising learning instead of giving a one-hour lecture. Specific topics in smaller chunks like 15 min is a better way for us to have a big picture of how things should be grouped and taught systematically.* BL08.

Concurrently, students could choose the optimal time of study to get the most out of learning. This further promotes better well-being as students who experienced blended learning approach felt less overwhelmed with the workload compared to their seniors who experienced the intensity of back-to-back classes across the week with traditional learning.

*My seniors last year told me that they have lecture from morning to afternoon. It’s really quite impossible for them to digest and absorb the information because the mind would just blank out and get so saturated with all those contents. This is not the case for us as we do not have such a packed schedule.* BL08.

The above illustrates how flexibility in learning enables a deeper approach to learning with intrinsic interest and maximised meaning behind the learning process. However, the inherent amount of workload in MBBS curriculum could lead to surface learning.

*There are lots of lecture information that you have to memorise. You never know… teachers might test this little information in exams.* BL09.

#### Instructional scaffolding to facilitate self-regulated learning in a blended learning approach

Students acknowledged significant self-regulated learning (SRL) occurring because of online learning. They reported that there is a need for faculty to create a well-designed learning environment. This includes clear learning sequence; specific learning outcomes that differentiates core versus supplementary knowledge; lecture delivery leveraging on clear text, visuals, and audio; supplementary learning resources for consolidation and application of knowledge; progress tracking mechanisms for students to monitor their learning.

*I think a general roadmap would be very helpful. Different lecturers employ different references and prioritise different things. It would be helpful to have content consistent across lectures. I also really appreciate Dr X’s efforts to prioritise things*,* for example*,* what is the core knowledge or foundation knowledge that every student must know. I think the greatest challenge…and need of support*,* would be how to integrate different lectures together… when you see a real patient presented to you.* BL06.

*Dr A is someone who’s adjusted really well in online learning format. Dr A has structured the content in a way that’s friendly to being viewed and learned on different devices in different settings… lots of pictures and the key points are all in the slides because sometimes we can’t hear Dr A well if we’re in traffic or wherever. Dr A has understood this and made a reasonable effort to address these challenges.* BL12.

*With online learning*,* it is quite hard to know our study progress and there is a need to track. We cannot compare with others*,* and we will be quite uncertain of how we are doing.* BL15.

Students report a need for a structured approach to support them in taking an active, self-directed role in their blended learning experience.

#### Cultivating interactions through blended learning

Students reported feeling isolated in the 2019/20 academic year when classes went fully online, despite the availability of online forums and live interactive sessions (on Zoom). They reflected that there are social norms for peer or student-teacher interactions. Peer communications often happen through informal channels (e.g. Whatsapp, social media, study groups, small groups). Student-teacher communications often happen via direct email. Several students noted they avoid engaging in online forum discussions due to a lack of anonymity, insufficient incentives for participation, or personal learning preferences. This suggests the need to create more engaging, incentivised, and personalised online discussion environments to foster the social connections that are typically present in face-to-face settings.

*We can’t really meet other students in-person. So*,* it feels like I’m learning alone…. I will not ask other students because I am not that close with them yet…. For communication with peers*,* it is usually Whatsapp or Instagram. And with teachers*,* it will be through email.* BL20.

*For questions on learning content*,* presently I think it would be quite embarrassing to message the whole group… because it might seem stupid to post some questions that might be obvious to others but isn’t obvious to myself.* BL04.

*For students to transition from just asking questions to engaging in online peer discussions*,* it is a habit that needs to be built over time. We still have the high school mentality that all answers need to be validated by teachers.* BL16.

In the subsequent 2020/21 academic year, several changes were implemented to cultivate more meaningful online interactions. Online forums were moderated by teachers and senior students. Automated notifications ensured teachers were aware of student questions. A reward system was used to incentivised active forum engagement. When social distancing measures eased, students were able to meet in-person for live sessions. Students emphasised that these face-to-face interactions were crucial for building the rapport necessary to support productive online discussions. Across interviews, students highlighted that cultivating a mutually supportive learning environment, with rapport-building between teachers, near-peers (seniors), and students, was more effective than relying on extrinsic rewards alone in promoting sustained online interactions.

*I am really happy to see students answering students’ questions and having further validation from the lecturer…. For seniors’ comments*,* they’re also really helpful because the seniors know exactly which point we’re stuck at. So*,* they can help us solve it straightforwardly*,* because they understand what the problem is as most of them have similar problems when they were studying in the previous years. The reason why students participated in forum is because they really want to learn*,* not really for the reward badges (extrinsic motivation).*

*We need more time to develop a relationship through this whole experience and physical exposure is better for us to know people and build rapport.* BL08.

*If we were to collaborate more on the online platform*,* we have to kind of get to know each other better.* BL13.

This suggests a multi-faceted approach combining technology-enabled moderation, notification systems, and in-person relationship building is key to fostering vibrant online learning communities.

#### Blended learning enables development of relevant skills, knowledge, and attitudes

Students reflected on the varied skills, knowledge, and attitudes acquired throughout their studies. Some skills and knowledge acquired are the understanding of disease manifestations and the relevant clinical interventions; infection control and public health measures (especially related to pandemic control); critique and evaluate scientific knowledge; choice of medication and contraindications. Examples of attitudes developed over time are the ethics and professionalism of a good doctor; lifelong learning; transdisciplinary and interprofessional collaboration.

Interestingly, some students reflected their perspectives of what makes a good educator in relation to their own studies and role as a future educator. Some expressed a desire to co-create the medical curriculum alongside the Faculty.

*From the curriculum*,* I can really learn a lot about what makes it good and what makes a bad teacher. I can learn from that and be a good teacher myself.* BL13.

*Even as a student*,* I’d be happy to assist with developing the curriculum because I have a stake in this. If I am taught well*,* I will learn well.* BL12.

## Discussion

Through deep learning, students learn with the intention of understanding and constructing meaning for critical thinking, application, and integration of knowledge. This stands in contrast to surface learning, which is characterised by rote memorisation and acquisition of knowledge to complete tasks or pass assessments. Our in-depth interviews brought clarity to parts of the curriculum which promoted deep or surface learning.

The in-depth interviews revealed that students gravitated towards deep learning with blended learning, as the online learning component provided time and space for full content comprehension, while face-to-face sessions consolidated and integrated that learning. However, postgraduate students with established learning preferences may favour traditional classroom learning at certain stages but acknowledge online learning as a supplement for didactic teaching. Quantitative and qualitative data corroborated that blended learning significantly improved clarity, understanding, application, and intrinsic motivation. These were enabled by its multi-modal features catering to diverse learner needs [30–33]. Yet, students also recognised that the self-directed nature of this blended approach necessitated clear instructional scaffolding to guide the learning process effectively [34].

Quantitative data (Fig. 4) indicated that students undertaking blended learning generally perceived a more manageable workload compared to face-to-face instruction. However, in-depth exploration of Year 2 students’ lived experiences and R-SPQ-2F data revealed an increasing loss of deep learning, regardless of whether traditional learning or blended learning was employed. Further investigation uncovered that Year 2 curriculum was densely packed with conceptual learning, research projects, and community engagement activities, which students reported as overwhelming. This finding aligns with existing research demonstrating that having a positive academic emotional response is a crucial mediator of deep learning in online environments [21]. Academic emotions are positive or negative emotions generated in the academic process, affecting learning motivation, strategies and performance [35, 36]. Specifically, the students described feelings of being overwhelmed by the workload, confused about the scope of learning, and anxious about examinations. These seemed to have negatively impacted their learning processes, leading to a greater reliance on surface-level strategies (Fig. 3B and D).

Social interactions are also important for advancing the learning process and internalising knowledge, thereby encouraging deeper learning approaches [34, 37, 38]. Our study shows that teacher-student interactions were perceived as adequate via feedback obtained in self-assessments and forums. This is pedagogically relevant as student-teacher interactions within a large student cohort were often perceived to be inadequate. Our blended learning approach enabled these interactions through automated feedback via online self-assessments, and feedback equitable to whole cohort via forums.

However, student-student (peer) interactions were significantly lacking through blended learning. In the online learning setting, effective peer interaction is much more difficult to achieve due to the various online communication media made available [39]. Nevertheless, our interviews revealed that prior face-to-face learning enabled rapport building among peers, which was then necessary for subsequent communication and collaborative peer feedback across online and in-person avenues. This aligns with Wang and colleagues’ research, which underscored the importance of establishing good interpersonal relationships before incorporating substantive peer interaction into a course [40]. Our findings further highlight the need to prioritise intrinsic motivation over extrinsic motivation when designing communication platforms that are equitable to the whole cohort [41]. From our data and previous reports, we found that this can be achieved through near-peer support, increased accessibility to forums, immediate and personalised learning feedback [42].

Figure 4 demonstrated that blended learning enabled the development of skills and knowledge, like traditional learning. This was further verified through the interviews, when different students reflected development of a range of skills, knowledge, and attitudes essential for their professional career. Interestingly, some students expressed a desire to actively co-create the medical curriculum alongside the faculty, reflecting a growing sense of partnership in shaping medical education.

### Limitations and strengths

A limitation of the study is the sample size for the quantitative evaluation, as participation from students were completely voluntary. However, we further employed a mixed-method approach that triangulated the quantitative data with 22 in-depth interviews that strengthened the study. This mixed-method approach and the richness of the interview data enhanced the information power of the study. Furthermore, no data was available to evaluate the impact of traditional learning on Year 1 students. It was not ethically appropriate to potentially disadvantage a portion of a Year 1 cohort within the 2-year study period by exposing them to different learning approaches. Another further limitation is potential participant bias as the consenting participants could be inherently active learners, and thus provide a positive perception of their blended learning experience. Nevertheless, their insights are crucial towards building an optimal blended learning environment. Lastly, we were unable to report assessment data on knowledge and skill competencies, as this was confidential institutional data.

This present study has led to transformative changes in our medical curriculum, including the sustainability of the blended learning approach, mutually supportive learning environments, as well as interactive face-to-face sessions that enable integration and application of knowledge. We believe that this study adds valuable insights to the medical education literature on opportunities and challenges in sustaining deep learning within a blended learning environment.

Moving forward, practical recommendations that are generalisable in higher education settings can be made from this study which promotes an optimised blended learning model. First, the multi-modality of online and/or face-to-face learning can be fully utilised to accommodate learner needs and differences. Second, online learning provides learning flexibility that can maximise deep learning, if the curriculum workload is primarily manageable. Third, instructional scaffolding is necessary to empower students for self-directed learning. Finally, building effective communication, collaboration and feedback processes that facilitate deep learning requires both technology-enabled moderation and in-person rapport building.

## Conclusions

Our research findings reinforce the notion that a well-designed learning approach can facilitate deep learning motives and strategies. Specifically, the blended learning model accommodates diverse learner needs and preferences through integration of multiple modalities. This can lead to a more manageable workload and affords students the flexibility to self-regulate their pace of study. These features of blended learning are instrumental in enabling deep approaches to learning. However, the self-directed nature of blended learning necessitates appropriate instructional scaffolding and support from educators. Communication and collaboration, which are critical elements of deep learning and contribute to feedback processes, were challenging to achieve through asynchronous and virtual means. Our study reports that communication and collaboration can be cultivated by the intentional development of a mutually supportive learning environment via technology-enabled moderation and in-person rapport building.

## Electronic supplementary material

Below is the link to the electronic supplementary material.

Supplementary Material 1. Additional file 1: R-SPQ-2F questionnaire.

Supplementary Material 2. Additional file 2: LE questionnaire.

Supplementary Material 3. Additional file 3: Interview guide.

Supplementary Material 4. Additional file 4: Participants’ characteristics according to SAL theory.

## Data Availability

The datasets generated and/or analysed during the current study are not publicly available due to ethics limitation but are available from the corresponding author on reasonable request.

## Abbreviations

HKUMed: The University of Hong Kong Medical Faculty
R-SPQ-2F: Revised two-factor study process questionnaire
LE: Learning experience
MOOC: Massive Open Online Learning Course
T&L: Teaching and learning
HPE: Health professions education
SAL theory: Student Approaches to Learning theory
MBBS: Bachelor of Medicine and Bachelor of Surgery
COVID-19: Coronavirus disease 2019
DA: Deep Approach
SA: Surface Approach
DM: Deep Motive
DS: Deep Strategy
SM: Surface Motive
SS: Surface Strategy
