# Supplementary material for: Surface and deep learning: a blended learning approach in preclinical years of medical school
Source: BMC Med Educ. 2024 Sep 19;24:1029. doi: 10.1186/s12909-024-05963-5 (PMC11414262; doi:10.1186/s12909-024-05963-5)
Supplement: Supplementary file 1 — Supplementary Material 1. Additional file 1: R-SPQ-2F questionnaire. [file 12909_2024_5963_MOESM1_ESM.pdf]

# PRE-COURSE R-SPQ SURVEY

**For Intervention & Control Group**

*This pre-course survey aims to collect participant's pre-course study-process characteristics* ***<u>prior to starting</u>*** ***HKU MBBS 1st year curriculum / 2nd year curriculum.***

## Revised Study-Process Questionnaire (10 min)

Please answer the questions below based on your study methods and experience before the start of this semester.

For each statement item, please choose one response accordingly: A – this item is *never* or *only rarely* true of me. B – this item is *sometimes* true of me. C – this item is true of me about *half the time.* D – this item is *frequently* true of me. E – this item is *always* or *almost always* true of me.

| | **Statement Item** | **Response (Circle ONE)** | | | | |
|---|---|---|---|---|---|---|
| 1 | I find that at times studying gives me a feeling of deep personal satisfaction. | **A** | **B** | **C** | **D** | **E** |
| 2 | I find that I have to do enough work on a topic so that I can form my own conclusions before I am satisfied. | **A** | **B** | **C** | **D** | **E** |
| 3 | My aim is to pass the course while doing as little work as possible. | **A** | **B** | **C** | **D** | **E** |
| 4 | I only study seriously what is given out in class or in the course outlines. | **A** | **B** | **C** | **D** | **E** |
| 5 | I feel that virtually any topic can be highly interesting once I get into it. | **A** | **B** | **C** | **D** | **E** |
| 6 | I find most new topics interesting and often spend extra time trying to obtain more information about them. | **A** | **B** | **C** | **D** | **E** |
| 7 | I do not find my course very interesting so I keep my work to the minimum. | **A** | **B** | **C** | **D** | **E** |
| 8 | I learn some things by rote, going over and over them until I know them by heart even if I do not understand them. | **A** | **B** | **C** | **D** | **E** |
| 9 | I find that studying academic topics can at times be as exciting as a good novel or movie. | **A** | **B** | **C** | **D** | **E** |
| 10 | I test myself on important topics until I understand them completely. | **A** | **B** | **C** | **D** | **E** |
| 11 | I find I can get by in most assessments by memorizing key sections rather than trying to understand them. | **A** | **B** | **C** | **D** | **E** |
| 12 | I generally restrict my study to what is specifically set as I think it is unnecessary to do anything extra. | **A** | **B** | **C** | **D** | **E** |
| 13 | I work hard at my studies because I find the material interesting. | **A** | **B** | **C** | **D** | **E** |

| | | | | | | |
|---|---|---|---|---|---|---|
| 14 | I spend a lot of my free time finding out more about interesting topics which have been discussed in different classes. | **A** | **B** | **C** | **D** | **E** |
| 15 | I find it is not helpful to study topics in depth. It confuses and wastes time, when all you need is a passing acquaintance with topics. | **A** | **B** | **C** | **D** | **E** |
| 16 | I believe that lecturers shouldn't expect students to spend significant amounts of time studying material that everyone knows won't be examined. | **A** | **B** | **C** | **D** | **E** |
| 17 | I come to most classes with questions in mind that I want answering. | **A** | **B** | **C** | **D** | **E** |
| 18 | I make a point of looking at most of the suggested readings that go with the lectures. | **A** | **B** | **C** | **D** | **E** |
| 19 | I see no point in learning material which is not likely to be in the examination. | **A** | **B** | **C** | **D** | **E** |
| 20 | I find the best way to pass examinations is to try to remember answers to likely questions. | **A** | **B** | **C** | **D** | **E** |

The responses to items are scored as follows:
A= 1, B = 2, C = 3, D= 4, E= 5

To obtain main scale scores add item scores as follows:
DA= 1 + 2 + 5 + 6 + 9 + 10 + 13 + 14 + 17 + 18
SA = 3 + 4 + 7 + 8 + 11 + 12 + 15 + 16 + 19 + 20

Subscale scores can be calculated as follows:
DM= 1 + 5 + 9 + 13 + 17
DS = 2 + 6 + 10 + 14 + 18
SM = 3 + 7 + 11 + 15 + 19
SS = 4 + 8 + 12 + 16 + 20

# POST-COURSE R-SPQ SURVEY

**For Intervention & Control Group**

*This post-course survey aims to collect participant's post-course study-process characteristics* ***<u>during</u>*** ***the HKU MBBS 1st year curriculum / 2nd year curriculum****.*

## Revised Study-Process Questionnaire (10 min)

Please answer the questions below based on your study methods and experience during the current semester.

For each statement item, please choose one response accordingly: A – this item is *never* or *only rarely* true of me. B – this item is *sometimes* true of me. C – this item is true of me about *half the time.* D – this item is *frequently* true of me. E – this item is *always* or *almost always* true of me.

| | **Statement Item** | **Response (Circle ONE)** | | | | |
|---|---|---|---|---|---|---|
| 1 | I find that at times studying gives me a feeling of deep personal satisfaction. | **A** | **B** | **C** | **D** | **E** |
| 2 | I find that I have to do enough work on a topic so that I can form my own conclusions before I am satisfied. | **A** | **B** | **C** | **D** | **E** |
| 3 | My aim is to pass the course while doing as little work as possible. | **A** | **B** | **C** | **D** | **E** |
| 4 | I only study seriously what is given out in class or in the course outlines. | **A** | **B** | **C** | **D** | **E** |
| 5 | I feel that virtually any topic can be highly interesting once I get into it. | **A** | **B** | **C** | **D** | **E** |
| 6 | I find most new topics interesting and often spend extra time trying to obtain more information about them. | **A** | **B** | **C** | **D** | **E** |
| 7 | I do not find my course very interesting so I keep my work to the minimum. | **A** | **B** | **C** | **D** | **E** |
| 8 | I learn some things by rote, going over and over them until I know them by heart even if I do not understand them. | **A** | **B** | **C** | **D** | **E** |
| 9 | I find that studying academic topics can at times be as exciting as a good novel or movie. | **A** | **B** | **C** | **D** | **E** |
| 10 | I test myself on important topics until I understand them completely. | **A** | **B** | **C** | **D** | **E** |
| 11 | I find I can get by in most assessments by memorizing key sections rather than trying to understand them. | **A** | **B** | **C** | **D** | **E** |
| 12 | I generally restrict my study to what is specifically set as I think it is unnecessary to do anything extra. | **A** | **B** | **C** | **D** | **E** |
| 13 | I work hard at my studies because I find the material interesting. | **A** | **B** | **C** | **D** | **E** |

| | | | | | | |
|---|---|---|---|---|---|---|
| 14 | I spend a lot of my free time finding out more about interesting topics which have been discussed in different classes. | **A** | **B** | **C** | **D** | **E** |
| 15 | I find it is not helpful to study topics in depth. It confuses and wastes time, when all you need is a passing acquaintance with topics. | **A** | **B** | **C** | **D** | **E** |
| 16 | I believe that lecturers shouldn't expect students to spend significant amounts of time studying material that everyone knows won't be examined. | **A** | **B** | **C** | **D** | **E** |
| 17 | I come to most classes with questions in mind that I want answering. | **A** | **B** | **C** | **D** | **E** |
| 18 | I make a point of looking at most of the suggested readings that go with the lectures. | **A** | **B** | **C** | **D** | **E** |
| 19 | I see no point in learning material which is not likely to be in the examination. | **A** | **B** | **C** | **D** | **E** |
| 20 | I find the best way to pass examinations is to try to remember answers to likely questions. | **A** | **B** | **C** | **D** | **E** |

The responses to items are scored as follows:
A= 1, B = 2, C = 3, D= 4, E= 5

To obtain main scale scores add item scores as follows:
DA= 1 + 2 + 5 + 6 + 9 + 10 + 13 + 14 + 17 + 18
SA = 3 + 4 + 7 + 8 + 11 + 12 + 15 + 16 + 19 + 20

Subscale scores can be calculated as follows:
DM= 1 + 5 + 9 + 13 + 17
DS = 2 + 6 + 10 + 14 + 18
SM = 3 + 7 + 11 + 15 + 19
SS = 4 + 8 + 12 + 16 + 20
