# Supplementary material for: Surface and deep learning: a blended learning approach in preclinical years of medical school
Source: BMC Med Educ. 2024 Sep 19;24:1029. doi: 10.1186/s12909-024-05963-5 (PMC11414262; doi:10.1186/s12909-024-05963-5)
Supplement: Supplementary file 2 — Supplementary Material 2. Additional file 2: LE questionnaire. [file 12909_2024_5963_MOESM2_ESM.pdf]

LEARNING EXPERIENCE QUESTIONNAIRE

For Intervention and Control Group

This questionnaire aims to understand student perception and experience of the MBBS learning approaches.

For intervention group, the 'current teaching and learning approach' mentioned in this survey includes mainly 3 components: Online video lectures with self-assessment questions; online discussion forum; face-to-face/live interactive session.

For control group, the 'current teaching and learning approach' describes the usual traditional classroom learning students experienced in some of their courses, which do not involve any of the e-Learning or blended learning approach elements.

Student perception of MBBS learning approach (10 min)

The following response scale will be used for each questions:
1 Strongly disagree
2 Disagree
3 Neither agree nor disagree
4 Agree
5 Strongly agree

1. Teaching material provided in the lecture was clear and easy to understand.
2. Teaching material provided in the lecture was delivered at an appropriate pace and in a logical sequence.
3. The current teaching and learning approach enabled me to develop skills needed by professionals in this field (e.g. professional communication, decision making, clinical observations).
4. The current teaching and learning approach encouraged me to apply theories and principles in the practice of medicine.
5. The current teaching and learning approach stimulated my interest such that I do more outside reading on the subject.
6. The current teaching and learning approach stimulated and encouraged me to ask questions about issues and topics in this field.
7. The current teaching and learning provides good quality feedback.
8. The current teaching and learning approach provides good lecturer-student interactions.
9. The current teaching and learning approach provides good student-student interactions.
10. The current teaching and learning approach allows me to develop autonomy in learning.
11. The current teaching and learning approach has a manageable workload.
