# Supplementary material for: Surface and deep learning: a blended learning approach in preclinical years of medical school
Source: BMC Med Educ. 2024 Sep 19;24:1029. doi: 10.1186/s12909-024-05963-5 (PMC11414262; doi:10.1186/s12909-024-05963-5)
Supplement: Supplementary file 3 — Supplementary Material 3. Additional file 3: Interview guide. [file 12909_2024_5963_MOESM3_ESM.pdf]

POST-COURSE SEMI-STRUCTURED INTERVIEW

For Intervention Group

General Focus on Advantages and Disadvantages of Blended Learning in MBBS Curriculum

1. In your opinion, what are the advantages of the blended learning approach in MBBS curriculum? Please elaborate, if appropriate.
2. In your opinion, what are the disadvantages of blended learning in MBBS curriculum? Please elaborate, if appropriate.

Learning Facilitators

3. What would be your suggestion to improve blended learning as a learning approach for MBBS curriculum?
4. Are the various components of blended learning helpful? Please specify the component and elaborate.
5. Given the big student cohort, how can we build effective peer interactions and collaboration?
6. In what types of MBBS courses might e-Learning be particularly valuable or less valuable?
7. In your opinion, what should be the proportion of e-Learning and face-to-face learning in MBBS curriculum? Please explain why.

Learning Challenges

8. What are the current learning challenges faced in the MBBS curriculum?

Outcomes

9. What are the newly acquired skills, knowledge or attitude learnt from the curriculum? Are these skills, knowledge, or attitude used in your everyday environment? If yes, then how so?

Open Discussion

10. Do you have any other comments?
