# Supplementary material for: Surface and deep learning: a blended learning approach in preclinical years of medical school
Source: BMC Med Educ. 2024 Sep 19;24:1029. doi: 10.1186/s12909-024-05963-5 (PMC11414262; doi:10.1186/s12909-024-05963-5)
Supplement: Supplementary file 4 — Supplementary Material 4. Additional file 4: Participants’ characteristics according to SAL theory. [file 12909_2024_5963_MOESM4_ESM.pdf]

TABLE Participant's characteristics

Identifier	Prior online learning / e-Learning experience	Postgraduate / Undergraduate*	Preferred learning approaches during MBBS study
BL01	No	Undergraduate	Blended
BL02	No	Undergraduate	Blended
BL03	No	Undergraduate	Blended
BL04	No	Undergraduate	Fully e-Learning
BL05	No	Undergraduate	Blended
BL06	Yes	Postgraduate	Blended
BL07	Yes	Postgraduate	Blended
BL08	No	Undergraduate	Blended
BL09	No	Undergraduate	Blended
BL10	No	Undergraduate	Blended
BL11	Yes	Undergraduate	Fully e-Learning
BL12	No	Undergraduate	Blended
BL13	No	Undergraduate	Blended
BL14	No	Undergraduate	Blended
BL15	No	Undergraduate	Blended
BL16	Yes	Undergraduate	Blended
BL17	Yes	Undergraduate	Blended
BL18	Yes	Undergraduate	Blended
BL19	Yes	Postgraduate	Face-to-face
BL20	Yes	Undergraduate	Blended
BL21	Yes	Undergraduate	Blended
BL22	Yes	Undergraduate	Blended

*Some students joined the MBBS programme as postgraduates, upon completing an undergraduate degree whereas some would join the programme as an undergraduate.
